# Establishment and characterization of a competitive exclusion bacterial culture derived from Nile tilapia (*Oreochromis niloticus*) gut microbiomes showing antibacterial activity against pathogenic *Streptococcus agalactiae*

**DOI:** 10.1371/journal.pone.0215375

**Published:** 2019-05-03

**Authors:** Javier Fernando Melo-Bolívar, Ruth Yolanda Ruiz Pardo, Michael E. Hume, David J. Nisbet, Fernando Rodríguez-Villamizar, Juan F. Alzate, Howard Junca, Luisa Marcela Villamil Díaz

**Affiliations:** 1 Universidad de La Sabana, Faculty of Engineering, Campus Universitario del Puente del Común, Km 7 Autopista Norte de Bogotá, Chía, Cundinamarca, Colombia; 2 Universidad de La Sabana, Faculty of Engineering, Grupo de Investigación en Procesos Agroindustriales, Campus Universitario del Puente del Común, Km 7 Autopista Norte de Bogotá, Chía, Cundinamarca, Colombia; 3 United States Department of Agriculture, Agricultural Research Service, Southern Plains Agricultural Research Center, College Station, TX, United States of America; 4 Corporación Colombiana de Investigación Agropecuaria (Agrosavia), Centro de investigación Tibaitatá, Mosquera, Cundinamarca, Colombia; 5 Centro Nacional de Secuenciación Genómica- CNSG, Sede de Investigación Universitaria SIU, Grupo de Parasitología, Facultad de Medicina Universidad de Antioquia, Medellín, Colombia; 6 Microbiomas Foundation, Div. Ecogenomics & Holobionts, RG Microbial Ecology: Metabolism, Genomics & Evolution, Chía, Colombia; University of Hong Kong, HONG KONG

## Abstract

This study reports the characterization of the microbial community composition, and the establishment and dynamics of a continuous-flow competitive exclusion culture (CFCEC) derived from gut microbiomes of Nile tilapia (*Oreochromis niloticus*) specimens reared on aquaculture farms in Colombia. 16S rRNA gene amplicon Illumina sequencing was used to identify taxonomical changes in the CFCEC microbial community over time. The CFCEC was developed from adult tilapia from two farms in Colombia, and CFCEC samples were collected over two months. The pH varied from 6.25 to 6.35 throughout culturing, while anaerobic and aerobic cell counts stabilized at day 9, at 10^9^ CFU mL^-1^ and were maintained to day 68. A variation in the CFCEC bacterial composition was observed over time. *Cetobacterium* was the most abundant in the first two days and coincided with a higher CFCEC supernatant antimicrobial effect against the fish pathogen *Streptococcus agalactiae*. Antimicrobial activity against *S*. *agalactiae* disappeared by day 3. Changes in bacterial composition continued to day 33 with *Lactococcus* spp. becoming the most abundant member of the community. In conclusion, the study of the CFCEC from intestinal tract of Nile tilapia (*Oreochromis niloticus*) by 16S rRNA gene sequencing allowed identification of predominant bacterial genera in the continuous-flow competitive exclusion culture exhibiting antibacterial activity against the fish pathogen *Streptococcus agalactiae*.

## 1. Introduction

The world population has been in continuous growth and it is expected to reach more than 9.6 million by 2050 [1]. Therefore, the United Nations proposed, among their sustainable development goals, the objective to reduce malnutrition (Zero Hunger) in the world’s human population. To achieve this goal, food industries are expected to increase production. Among them, the fish industry hopes to increase production by 19% in 2024 [2]. Wild-captured fishes have reached their production limit, therefore, aquaculture is one of the best alternatives to cover the demand for fish products [3].

To ensure this increase profitability, it is necessary to intensify crop production and culture densities, which has generated a rise in fish mortality due to infectious agents [4,5], among which are bacteria (*Streptococcus agalactiae*, *Aeromonas hydrophila*, *Flavobacterium columnare* and *Edwardsiella tarda*), parasites (*Trichodina* spp., *Ichthyophthirius* spp., *Piscinoodinium* spp. and *Eimeria* spp.) and viruses (herpes-like tilapia larvae encephalitis virus, necrosis viral nervous system and Nile tilapia virus) [6,7]. To counter emergence of these infectious agents, there has been increased use of antibiotics to prevent diseases [8], resulting in adverse health effects such as the selection and rise of multidrug-resistant bacteria [9].

An alternative to the use of antibiotics is the administration of probiotic bacteria, which have shown benefits in aquaculture both for water use sustainability and for fish health and productivity. Among other benefits, probiotic bacteria can improve the immune response and disease resistance of fishes, and can promote enhanced growth [10]. There are evidences showing that a mixture of microorganisms offers synergistic probiotic properties [11]. In recent years, due to the complexity of the intestinal microbiota of humans and other animals, probiotics composed of more than one strain have been developed [12]. The competitive exclusion culture is a technique by which a mixture of microorganisms from a healthy host are developed and maintained in order to selectively exclude enteropathogens [13].

The majority of competitive exclusion cultures have been applied to poultry, pigs and humans [14–16]. In a study with early weaned pigs it was shown that the application of a competitive exclusion culture decreased the prevalence of *Salmonella choleraesuis*, [17]. Similarly, Genovese et al. [18] showed that a pig-derived competitive exclusion culture decreased the incidence of disease caused by *Escherichia coli* in newborn pigs when receiving the treatment at 12 and 24 hours of age. A commercial competitive exclusion culture derived from chickens demonstrated its effectiveness in two application methods (crop gavage or coarse spray) to reduce the *Salmonella* spp. colonization in one-day-old turkeys [19]. Aviguard, a freeze-dried chicken-derived competitive exclusion commercial product, prevented multidrug-resistant *Escherichia coli* intestinal colonization for about 2 weeks in one-day-old chickens [20]. A competitive exclusion culture derived from human feces demonstrated an *in vitro* elimination of vancomycin-resistant *Enterococcus faecium* at concentrations of 10^3^ to 10^6^ CFU mL^-1^ [21].

In the case of tilapia, Iwashita et al. [5] used a mixture of *Bacillus subtilis*, *Saccharomyces cerevisiae*, and *Aspergillus oryzae*, showing an association among these microorganisms in the intestine. That association allowed up regulation of immune responses and a reduction in mortality due challenge with *Aeromonas hydrophila* and *Streptococcus iniae*, as well as increased feed conversion ratio, although not improved the growth rate of tilapia. Furthermore, a commercial mixture of *B*. *subtilis*, *E*. *faecium*, *Lactobacillus reuteri* and *Pediococcus acidilactici* (Aquastart® Growout, Biomin GmbH, Getzersdorf, Austria), when applied continuously over six weeks, improved growth and enhanced tilapia intestinal immunological status by increasing the abundance of goblet cells and intraepithelial lymphocytes [22].

In addition to studies with probiotic bacteria, there have been studies in which probiotic treatments have been combined with prebiotics or enzymes to improve their effectiveness. For example, the combination of the probiotic *Bacillus licheniformis* with a prebiotic derived from yeast extract improved growth parameters and the feed conversion ratio in Nile tilapia (*O*. *niloticus*) [23]. Furthermore, the combined use of the exogenous enzymes phytase, protease, and xylanase and a probiotic containing *B*. *subtilis*, *B*. *licheniformis*, and *Bacillus pumilus* improved growth parameters (final weight, specific growth rate, feed conversion ratio, and protein efficiency ratio) and fiber degradation. Similarly, this mixture improved the digestion of indigestible non-starch polysaccharides and trypsin inhibitors, which may produce necrotic enteritis in Nile tilapia [24]. In Colombia, *Enterococcus*, *Myroides* and *Exiguobacterium* were isolated from Nile tilapia and increased the specific growth rate and the survival of tilapia in a challenged against *Edwardsiella tarda* [25].

As far as we know this is the first report of the development and characterization of a continuous-flow competitive exclusion culture derived from the tilapia gastrointestinal tract.

## 2. Materials and methods

### 2.1. Continuous-flow competitive exclusion culture (CFCEC)

Adult Nile tilapia (O. niloticus), a not protected species, were donated by commercial fish producers from two fish farms in Colombia; the Llanos Farm (Langostinos del Llano, Restrepo, Meta), located in a neotropical savanna grassland region (specimen weight 649.5 g, n = 6) and the Atlantic Farm (La Gran Canaria, Suan, Atlantic), located in a neotropical humid savanna of the Magdalena River basin region (average specimen weight 391.7 g, n = 6). Fish were euthanized by the use of tricaine methanesulfonate (300 mg l−1) (MS222, Sigma-Aldrich, St. Louis, USA) according to the protocol reviewed and approved by the ethics review board at Universidad de La Sabana N° 57 of 2016 and transported to the laboratory in containers filled with ice [26], the international ethical guidelines for experiments with animals was also followed according to Directive 2010/63/EU and following Colombian national government regulations “Permits for accessing genetic resources was issued by the Colombian Ministry of Environment Number 117, 26 of May, 2015 Otrosí 4”. External disinfection of the fishes was performed with 70% ethanol. The intestine was removed aseptically. The intestinal luminal contents were scraped into sterile tubes [27]. A fraction of the thoroughly mixed contents was used immediately as inoculum for the CFCEC. The remainder contents were stored at -20°C for Illumina sequence analysis.

The CFCEC was developed according to the method of Aguilar-Rivera et al. [28] with some modifications. Briefly, 10 mL of the combined and thoroughly mixed intestinal contents from fish from the two farms were incubated in 100 mL of tryptic soy broth (TSB; Scharlau, Barcelona, Spain) at anaerobic conditions (O_2_: below 1%; CO_2_: 9–13%) in anaerobic jar (2.5 L AnaeroJar, Oxoid, Hampshire, England). Culture were incubated overnight at 27°C overnight and used as the initial seed culture. A portion, 100 mL, of this seed culture was inoculated into 900 mL of TSB contained in a 2-L Bioflo III reactor vessel (New Brunswick Scientific Co., Edison, NJ) to initiate the batch culture (0 days of the CFCEC), with constant agitation at 200 rpm, 30 cm^3^ min^-1^ of oxygen-free CO_2_ and at 27°C. After 24 h, the continuous culture was started at a flow rate of 0.69 mL min^-1^, maintaining the conditions of agitation, temperature and supply of oxygen-free CO_2_.

### 2.2. Characterization of CFCEC

The CFCEC was monitored over time to determine when the culture established a stable microbiota consortium. Parameters monitored were pH, total anaerobic and aerobic cell counts, bacterial diversity assessments by denaturing gradient gel electrophoresis (DGGE) of the variable V3 region of the 16S rRNA gene amplicon and Illumina sequencing of amplicons of the V4 variable region of the 16S rRNA gene and antibacterial activity of CFCEC supernatant extracellular/released metabolites.

#### 2.2.1. Culture pH and total anaerobic and aerobic cell counts

The culture pH was determined during fermentation with the Bioflo III pH electrode. The total anaerobic and aerobic cell counts were enumerated on tryptic soy agar (TSA; Scharlau, Barcelona, Spain) incubated under anaerobic conditions or aerobic conditions at 27°C, respectively, at anaerobic conditions (O_2_: below 1%; CO_2_: 9–13%) in anaerobic jar (2.5 L AnaeroJar, Oxoid, Hampshire, England).

#### 2.2.2. Characterization of the CFCEC bacterial diversity by denaturing gradient gel electrophoresis (DGGE) and Illumina sequencing

DNA was extracted from initial samples (Atlantic farm and Llanos farm), the seed culture, day 0, 1, 2, 3, 4, 5, 6, 7, 9, 11, 12, 15, 17, 18, 21, 24, 28, 33, 35, and 40 days incubation of the CFCEC using the Stool DNA Isolation kit (Norgen, Thorold, ON, Canada) according to the manufacturer´s instructions. DGGE was run according with the methodology of Hume [29], using primers to conserved regions flanking the variable V3 region of the 16S rRNA gene amplicon (primer 2: 5´-ATTACCGCGGCTGCTGG-3´; primer 3: 5´-GCCCGCCGCGCGCGGCGGGCGGGGCGGGGGCACGGGGGGCCTACGGGAGGCAGCAG-3´. 8% (vol/vol) polyacrylamide gel (acrylamide-bisacrylamide ratio 37.5:1, Bio-Rad Laboratories; Richmond, CA) cast with 35 to 60% of urea-deionized formamide (Sigma) gradient was used to separate the amplicons. Bands were stained with SYBR Green I (Sigma) (1:10,000 dilution).

The sample of the two farms, days 0, 1, 2, 3, 5, 17, 33 of the CFCEC were selected to identify the bacterial composition variation by 16S rRNA gene amplicon Illumina sequencing. PCR primers 515F/806R with barcodes were used to sequence the highly variable V4 region of the 16S rRNA gene. PCR was carried out with the HotStarTaq Plus Master Mix kit (Qiagen, Germantown, MD, USA) with the following conditions: initial denaturation at 94°C for 3 min, 28 cycles at 94°C for 30 s, at 53°C for 40 s, and 72°C for 60 s, and final extension at 72°C for 5 min. Amplicons were confirmed using 2% agarose gel electrophoresis. The PCR products were sequenced at Molecular Research DNA Lab (Shallowater, TX, USA) using 300 to 570 bp paired-end sequencing with an Illumina Miseq following the manufacturer´s guidelines.

Farms and CFCEC datasets obtained from the Illumina sequencing were analyzed with the Mothur (https://www.mothur.org/wiki/MiSeq_SOP) protocol proposed by Kozich et al. [30]including default parameters for paired-end read assembly, reduction in the number of redundant sequences, alignment of sequences to reference databases, removal of chimeric and non-16S sequences, picking the operational taxonomic units (OTU), taxonomy assignment, building of the OTU table and alpha diversity analyses. The SILVA 132 16S rRNA gene non-redundant database was used for the sequence alignments and classifications. The datasets were submitted to the Sequence Read Archive (https://www.ncbi.nlm.nih.gov/Traces/study/?acc=PRJNA493858).

#### 2.2.3. Culture supernatant antibacterial analysis by agar diffusion

The antibacterial activity of the CFCEC supernatant was evaluated using the well diffusion method [31]. Briefly, the CFCEC was sampled at 0 (as specified above), 1, 2, 3, 4, 5, 6, 7, 8, 9, 11, 12, 13, 14, 15, 17, 18, 24, 28, 33, 35, 42, 45, 54, 56 days and samples were centrifuged in sterile 1.5-mL microfuge tubes at 8,000 x g for 10 min. After 48 h of incubation at 27°C in TSB, *S*. *agalactiae*, representative of clonal outbreaks in Nile tilapia cultures in Colombia [32], was adjusted to approximately 1.1 × 10^7^ CFU mL^-1^, and was used to inoculate TSA by streaking evenly across the surface using a sterile cotton swab. After 20 min, an 8-mm hole was made in the TSA using a sterile pipette tip and 100 μL of the CFCEC supernatant from the different incubation times was pipetted in triplicate holes and incubated 24 hours at 27ºC. Finally, according with Shokryazdan et al. [31], the inhibition was determined as the area around the hole where the bacteria have not growth enough to be visible.

### 2.3. Statistical analysis

Online DMfit (https://browser.combase.cc/DMFit.aspx) was used to model the growth parameters (initial value, final value, maximum growth rate) of the aerobe and anaerobe cell counts during CFCEC incubation. The Baranyi and Roberts predictive primary model defined by Eq (1) was used to determine when the microorganisms reached a plateau (stable microbiota consortium) [28]. To evaluate the goodness of fit of the predictive models, the following criteria were taken into account: standard error (SE), correlation coefficient (R2) and analysis of the estimates of the kinetic parameters [28]

$$y(t)=y_{0}+\mu_{max}A(t)-\frac{1}{m}\ln\left(1+\frac{e^{m\mu maxA(t)}-1}{e^{m(\gamma max-\gamma 0)}}\right)$$ (1)

where: *y*0 = *ln x* (*t*_0_): natural logarithm of the cell concentration at *t* = *t*_0_. *γmax* = *ln xmax*: natural logarithm of the maximum cellular concentration. *A* (*t*): function for the gradual delay in time (Lag phase). μ*max*: maximum growth rate.

DGGE fragment pattern relatedness was determined with molecular analysis fingerprinting software, version 1.6 (Bio-Rad Laboratories, Hercules, CA), based on the Dice similarity coefficient and the unweighted-pair group method using arithmetic averages for clustering [29].Alpha-diversity (Shannon diversity index, and Inverse Simpson estimators) was calculated using Mothur package. Non-metric multidimensional scaling (NMDS), which was used to see the ordinal distances between samples in two dimensions base on matrix, was performed in Mothur using the pipeline of MetaAmp [33]. The pearson correlation test was used to analyze the correlations between relative abundance of OTUs in the CFCEC and the antibacterial activity of the extracellular product of the CFCEC during the fermentation time in the statistical software SAS (version 9.4, Cary, NC, USA).

## 3. Results

### 3.1. Characterization of CFCEC

#### 3.1.1. Culture pH and total anaerobic and aerobic cell counts

**Fig 1:**
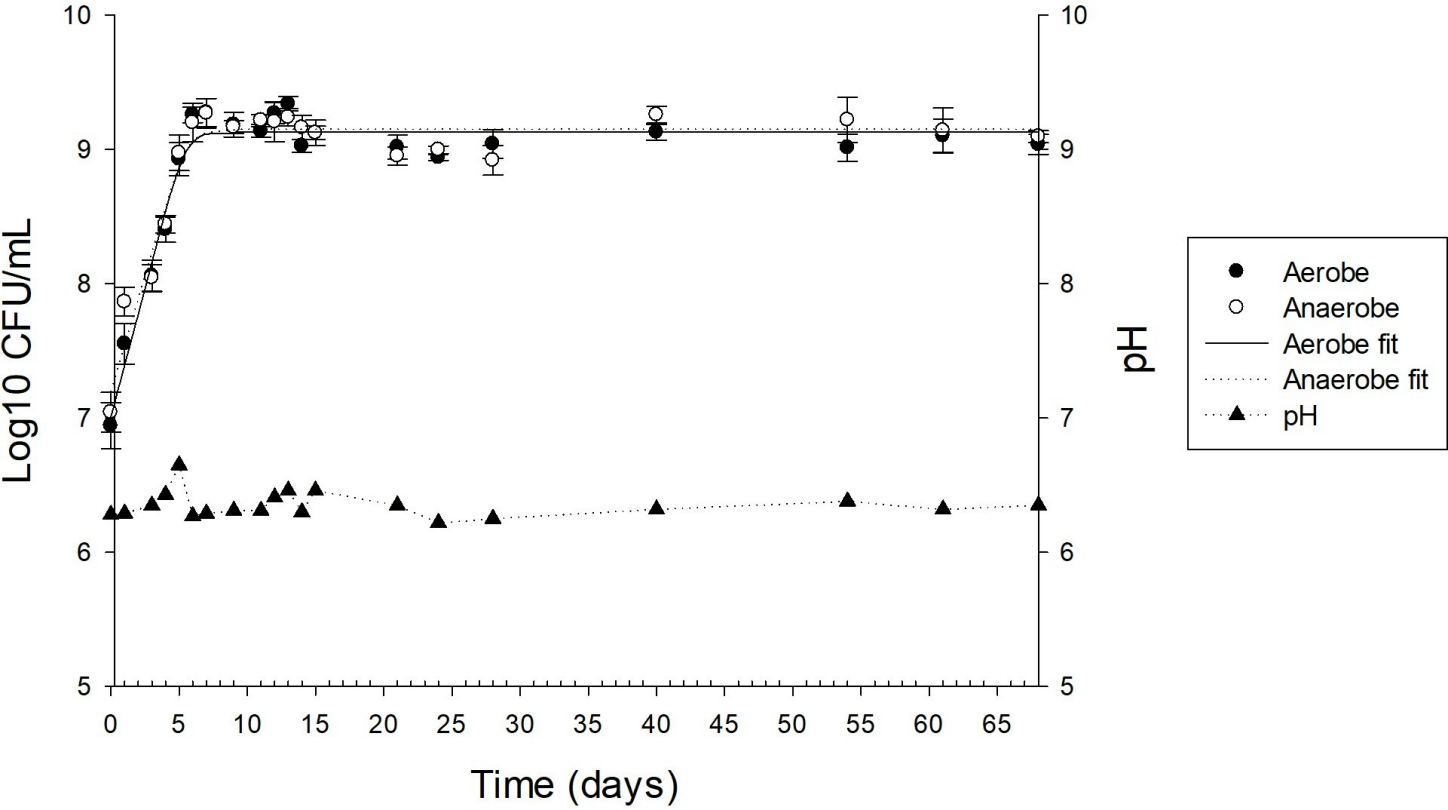
Nile tilapia (*Oreochromis niloticus*)-derived continuous-flow competitive exclusion culture bacterial cell counts for aerobes (●) and anaerobes (○), and fitting data to DMFit online-version in the primary y-axis, and pH (▲) in the secondary y-axis. Each symbol represents a mean ± SD of triplicate results.

The Baranyi and Roberts predictive primary model indicated that aerobe and anaerobe growth parameters were similar, with an initial count (initial value) of around 10^7^ CFU mL^-1^ and a final count (final value) of more than 10^9^ CFU mL^-1^ until day 68 and a maximum growth rate of less than 0.386 day^-1^ (Fig 1 and Table 1). The pH was stable, without external adjustments, between 6.25 to 6.35 during the 33 days evaluated.

**Table 1:** Aerobic and anaerobic bacteria growth parameters in a Nile tilapia-derived continuous-flow competitive exclusion culture.

	Initial Day 0 Log_10_ CFU/mL^1^	Final Day 68 Log_10_ CFU/mL	Maximum Rate (day^-1^)	SE of Fit	R-square
Aerobe	7.01 ± 0.10	9.13 ± 0.03	0.386 ± 0.035	0.129	0.957
Anaerobe	7.20 ± 0.12	9.15 ± 0.04	0.346 ± 0.039	0.149	0.935

^1^Values are means ± S.D., n = 4

#### 3.1.2. Characterization of CFCEC bacteria

**Fig 2:**
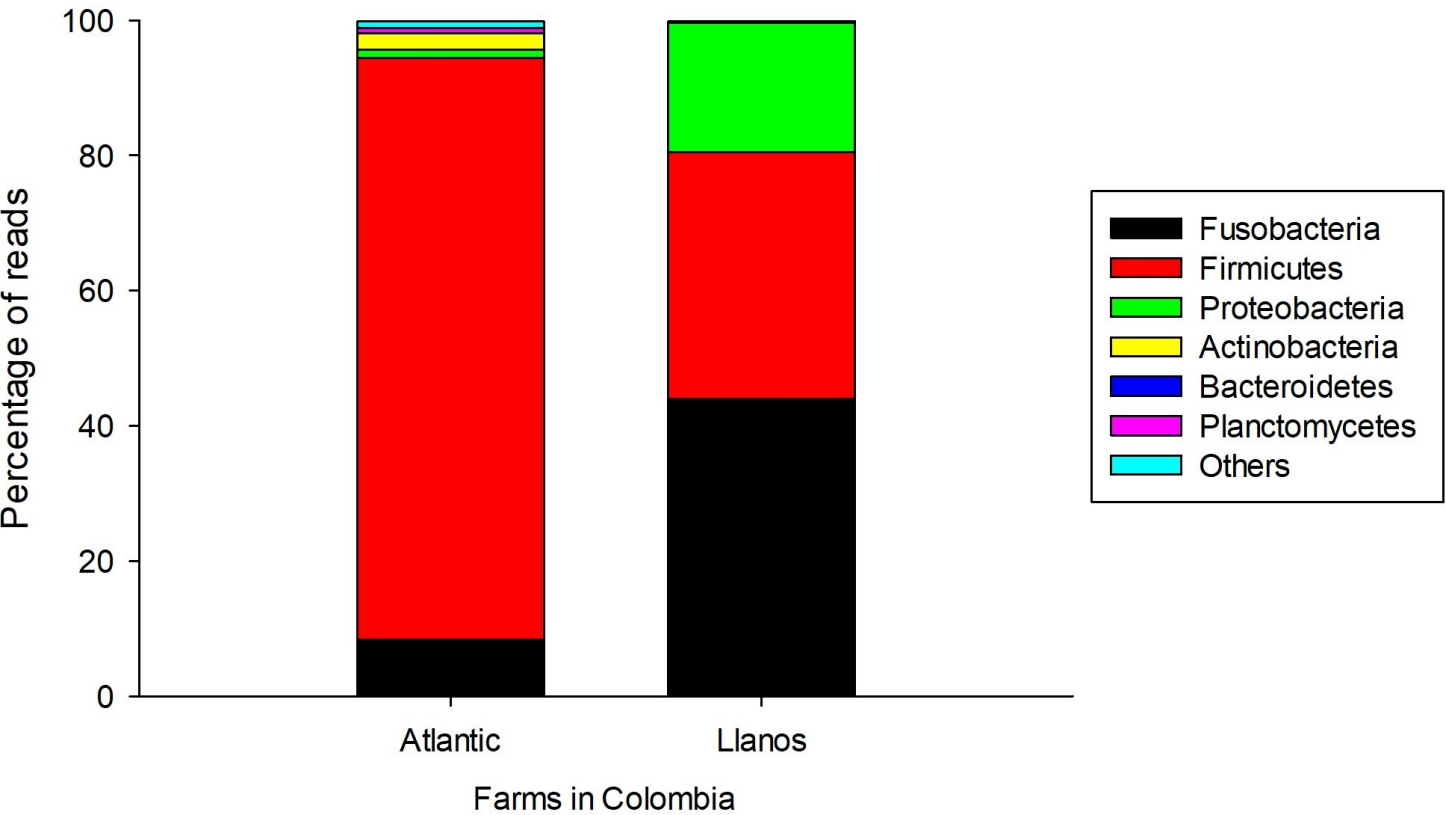
Relative abundance of intestinal bacteria phyla of Nile tilapia (*O*. *niloticus*) from two Colombian aquaculture farms (Atlantic and Llanos).

**Fig 3:**
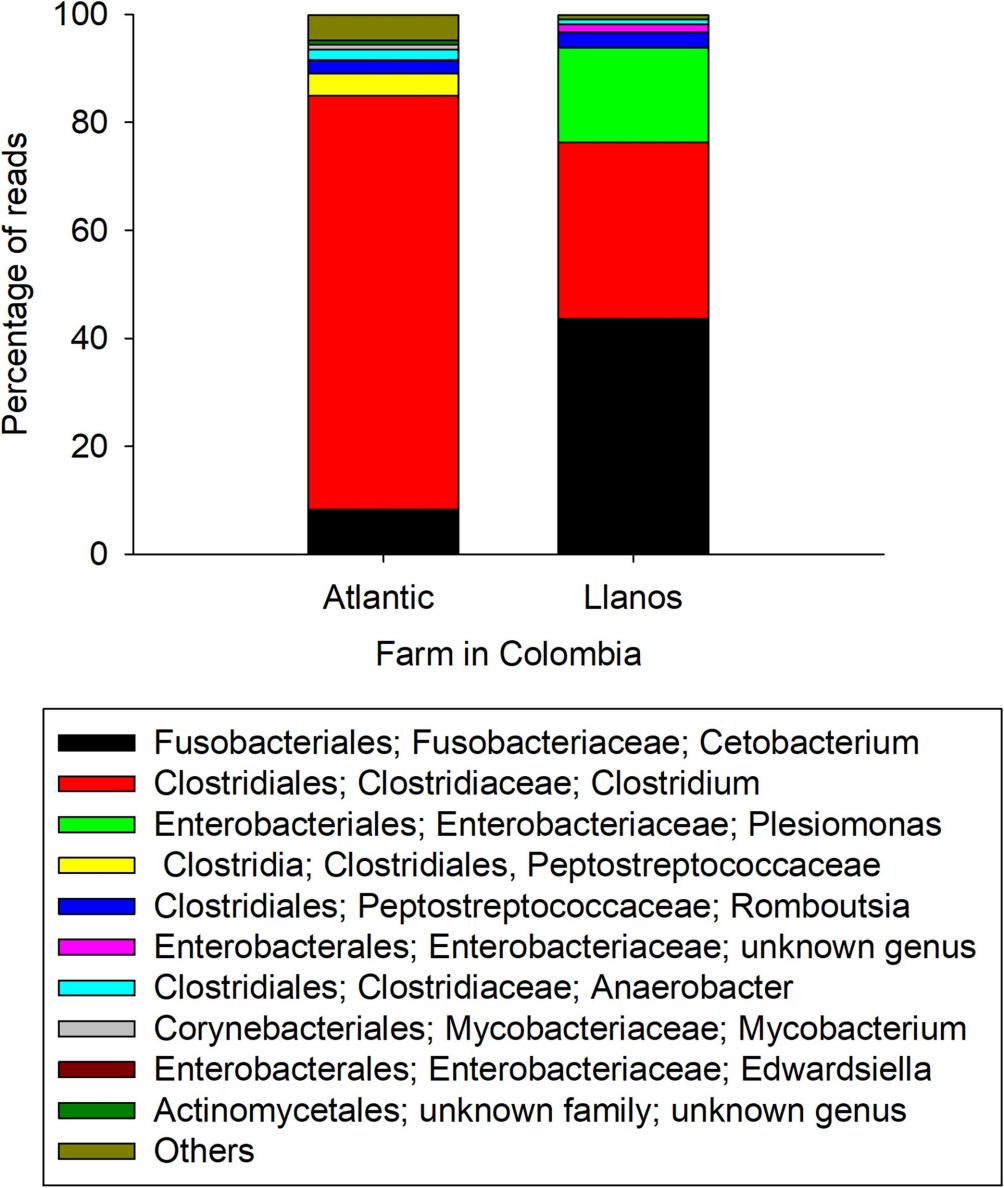
Relative abundance of intestinal bacteria genera of Nile tilapia (*O*. *niloticus*) from two Colombian aquaculture farms (Atlantic and Llanos).

The bacterial taxonomic composition of the Nile tilapia intestinal contents from the farms and from different days of the CFCEC was characterized by Illumina sequencing. The sample datasets ranged from 81,260 to 136,023 sequences, with more than 70% of the sequences passing the quality selection thresholds, not having chimeric sequences or non-16S sequences detected. The intestinal contents from the two farms had differences in bacterial composition. In Nille tilapia from the Atlantic farm, the most abundant phylum was Firmicutes and genus *Clostridium*. In contrast, for the Nile tilapia from the Llanos farm, the most abundant genus was *Cetobacterium* from the phylum Fusobacteria followed by *Clostridium* and *Plesiomonas* (Figs 2 and 3).

**Fig 4:**
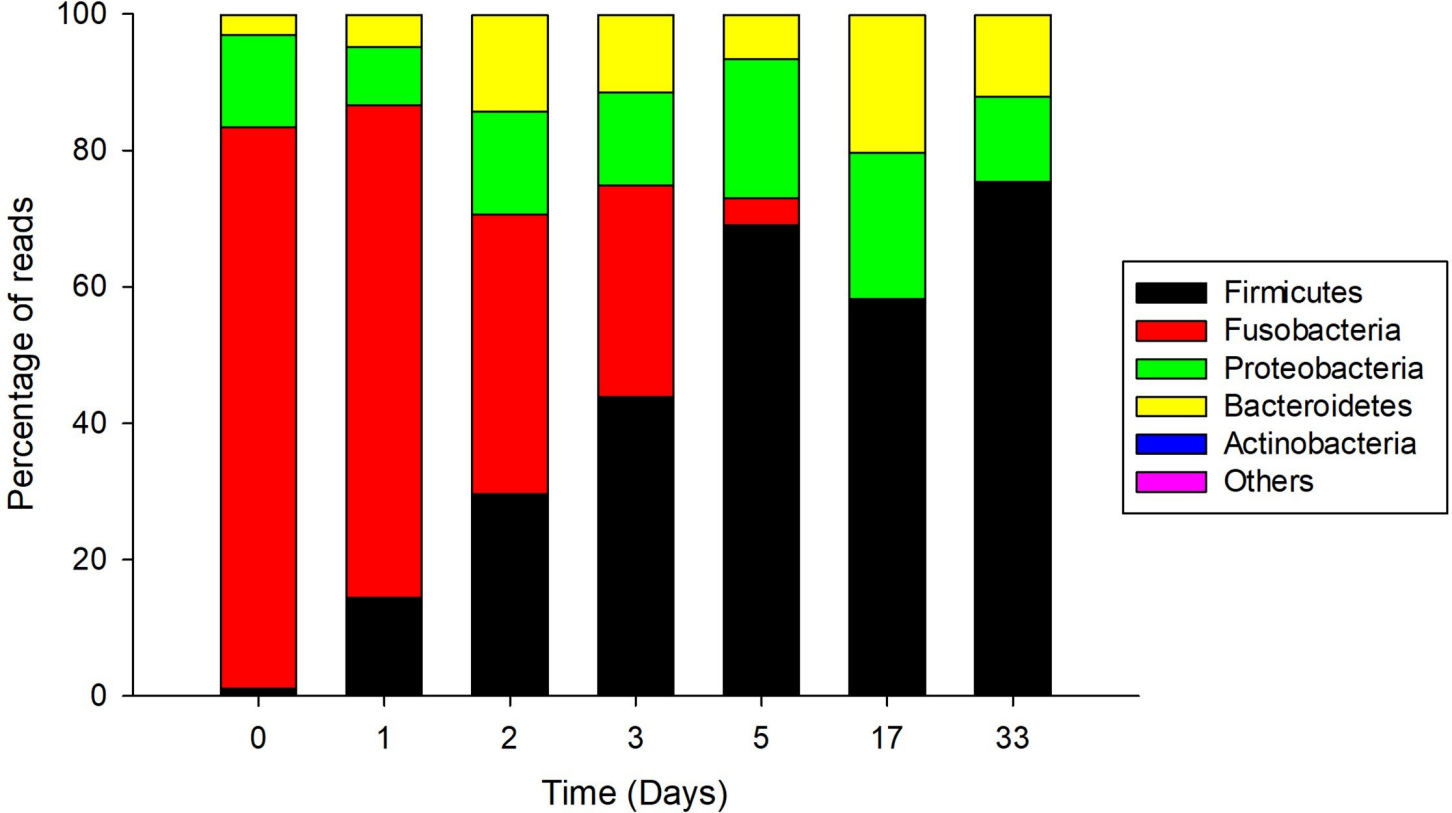
Relative abundance of intestinal bacteria phyla in a Nile tilapia (*Oreochromis niloticus*)-derived continuous-flow competitive exclusion culture.

**Fig 5:**
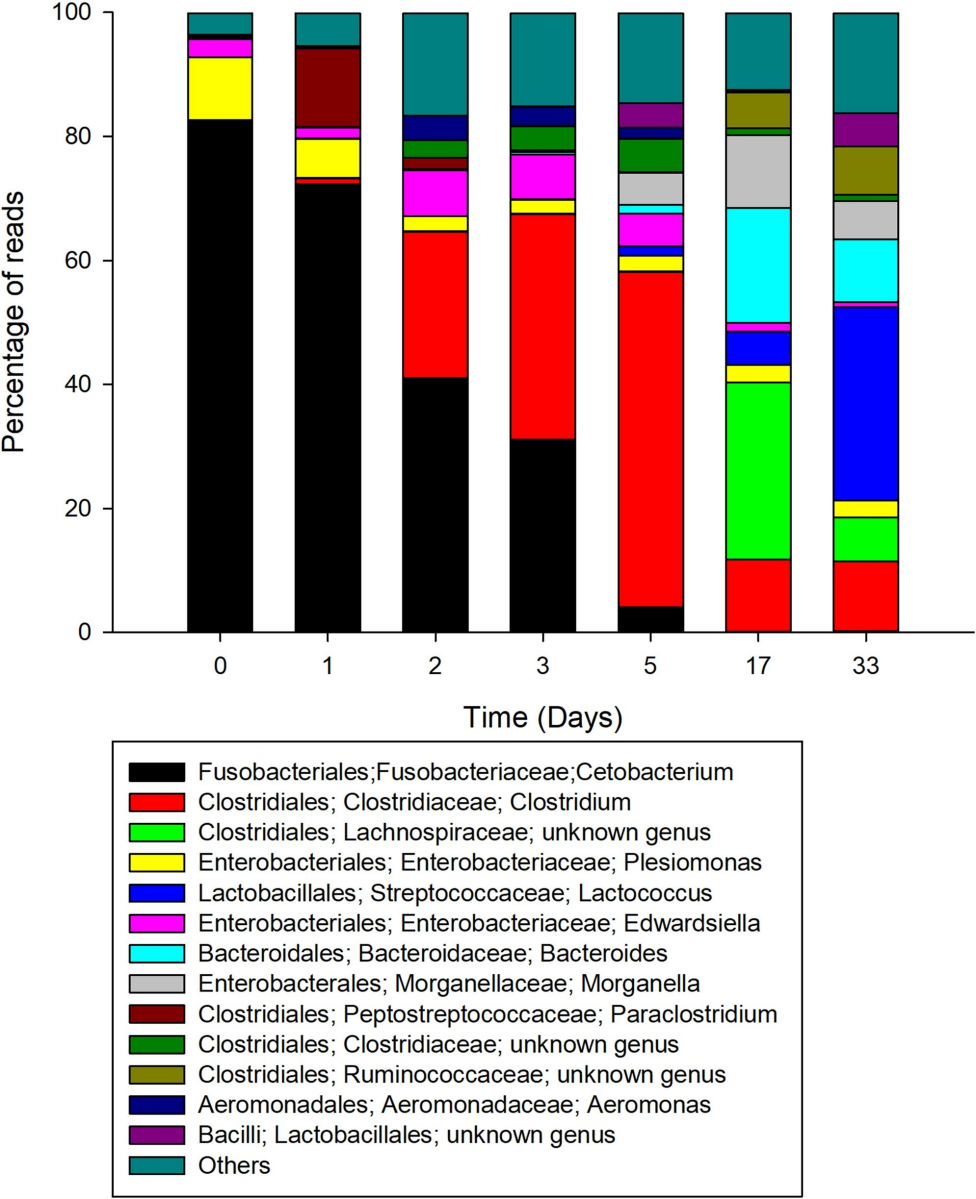
Relative abundance of intestinal bacteria genera in a Nile tilapia (*Oreochromis niloticus*)-derived continuous-flow competitive exclusion culture.

With regard to the CFCEC, the most abundant genera identified during the different stages were *Cetobacterium*, *Clostridium* and *Lactococcus*. Fusobacteria, with 80% of the sequences, was the most abundant phylum when the culture was inoculated with the seed (day 0) and the phylum Firmicutes at the 33 days of the CFCEC reach a high percentage of reads (Fig 4). At the genus level, *Cetobacterium* was the most abundant in the first two days after the start of the CFCEC, reaching more than 80% of the abundances in the first day. From day two, the *Clostridium* group abundance was increasing to approximately 40% by day 5, after which its abundance dropped to less than 15%. *Lactococcus* was the most abundant at day 33 of the CFCEC according to Illumina sequencing analysis (Fig 5).

The alpha diversity indices (Shannon and Inverse Simpson Indexes) were determined with Mothur. According to the Shannon and Inverse Simpson indexes, the first days of the reactor had less diversity with values slightly below 1.5 and 2.5, respectively, and from day 2 the Shannon index reach 2.4 and the Inverse Simpson 7 (Table 2).

**Table 2:** Changes in the Shannon diversity index and Inverse Simpson diversity Index for bacteria in the intestinal contents of Nile tilapia from two farms and the continuous-flow competitive exclusion culture (CFCEC).

Sample		Shannon diversity index	Inverse Simpson diversity index
Farm	Atlantic	2.69	5.67
	Llanos	2.16	4.32
Days of the CFCEC	0	1.17	1.99
	1	1.47	2.56
	2	2.37	7.23
	3	2.36	6.66
	5	2.41	5.40
	17	2.46	6.88
	33	2.57	7.33

**Fig 6:**
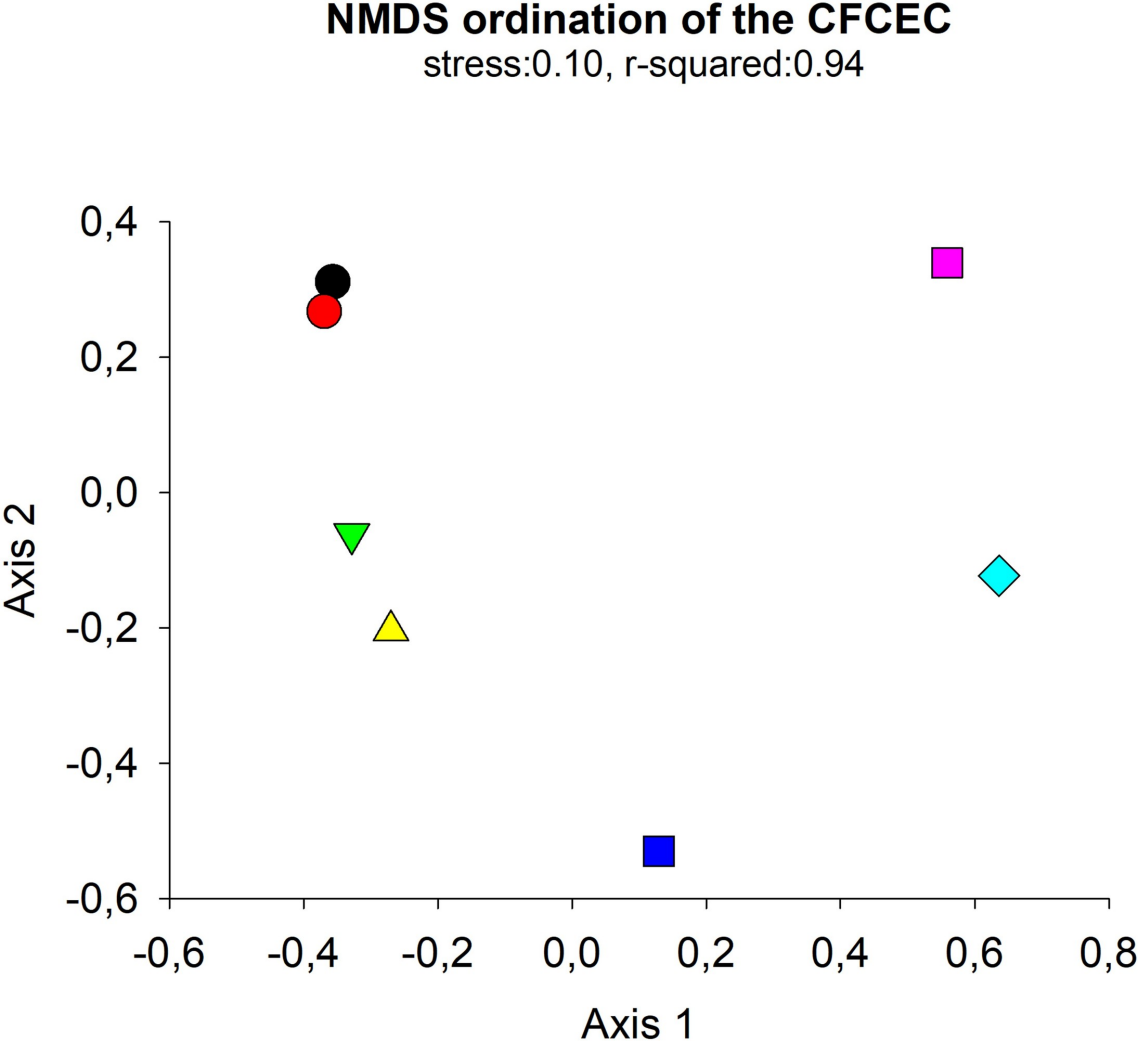
NMDS ordination derived from a thetaYC distance matrix of the bacterial 16S rRNA gene amplicon Illumina sequencing. Each point corresponds to the microbial community of the days 0, 1, 2, 3, 5, 17 and 33 of the CFCEC.

**Fig 7:**
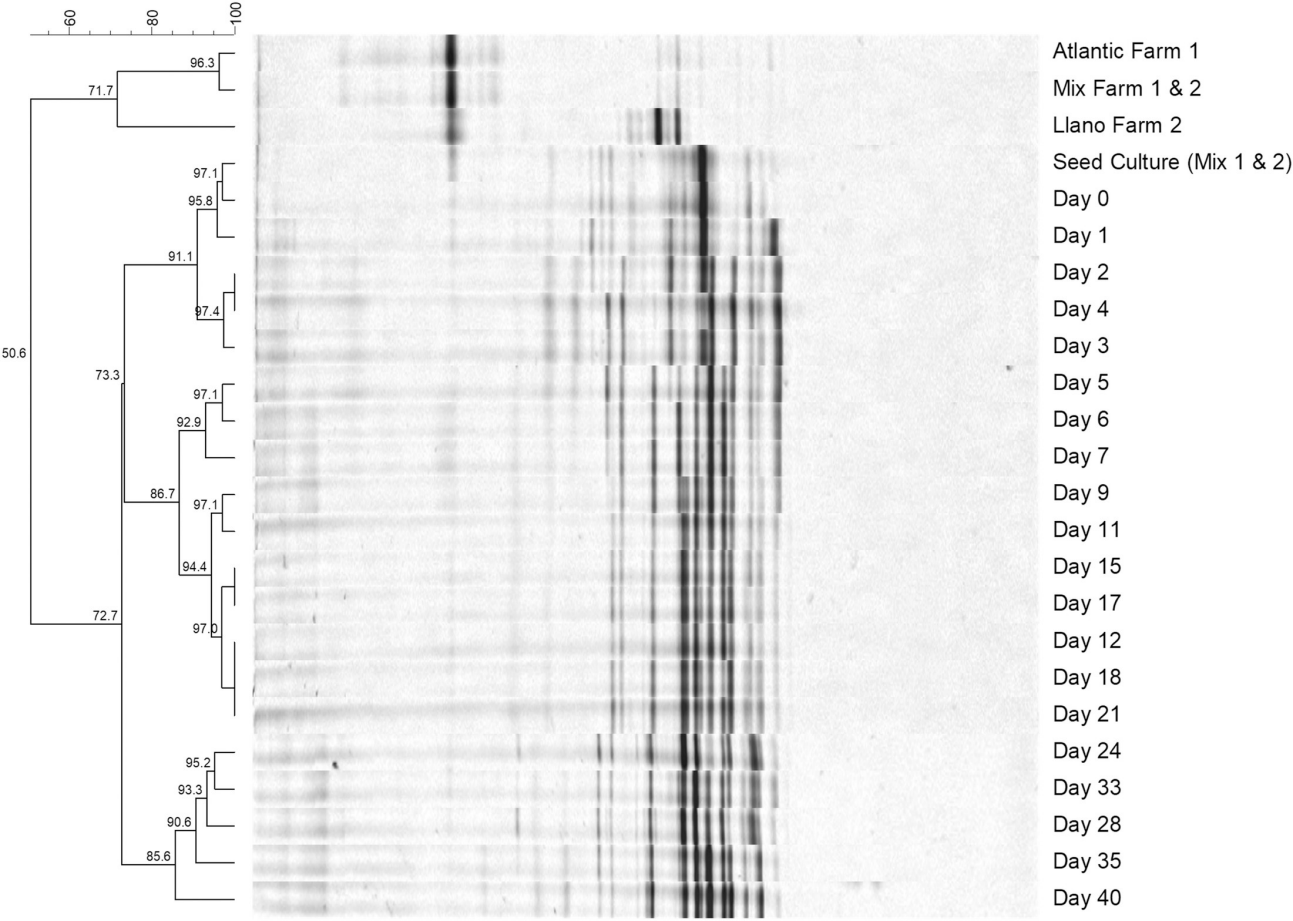
Denaturing gradient gel electrophoresis (DGGE) profiles of PCR-amplified bacterial gene fragments for the farms (Atlantic and Llanos) and the continuos-flow competitive exclusion derived from Nile tilapia (*Oreochromis niloticus*) gut microbiomes.

Non-metric Multi-Dimensional scaling (NMDS) of thetaYC distance matrix of the bacterial 16S rRNA gene amplicon Illumina sequencing was used to determine whether there were any significant differences between microbial composition between the days 0, 1, 2, 3, 5, 17 and 33 of the CFCEC. The NMDS demonstrated a clear separation of the data between the CFCEC samples showing five group during the fermentation (group 1: days 0,1; group 2: days 2, 3; group 3: day 5; group 4: day 17; group 5: day 33) (Fig 6). Those results were similar to the DGGE analysis, which show the same five groups of bacteria compositions (group 1: days 0,1; group 2: days 2, 3, 4; group 3: day 5, 6, 7, 9; group 4: day 11, 12, 15, 17, 18, 17, 21; group 5: days 24, 28, 33, 35, 40) (Fig 7).

#### 3.1.3. Culture medium antibacterial activity analysis by agar diffusion

Medium was collected and frozen from different times of the CFCEC and the antibacterial activity of the extracellular products was evaluated against *S*. *agalactiae* by the agar diffusion method. Antibacterial effect against *S*. *agalactiae* was detected from day 0 to 2 with an inhibition zone of 25 mm approximately and no inhibition zones were visualized over days 3 to 56 (S1 and S2 Figs). A positive pearson correlation showed that *Cetobacterium* and *Plesiomonas* (0.928 and 0.77, respectively) are directly related with the antibacterial activity of *S*. *agalactiae* from day 0 to 3 with a p-value > 0.05 (Table 3).

**Table 3:** Analysis of the pearson correlation to identify the change in bacterial abundance of each genus in the CFCEC with respect to the antibacterial activity.

Bacteria	Pearson correlation (p-value*)
**Fusobacteriales; Fusobacteriaceae; *Cetobacterium***	**0.92805**
	**(0.0026)**
**Clostridiales; Clostridiaceae; *Clostridium***	-0.63352
	(0.1266)
**Clostridiales; Lachnospiraceae; unknown genus**	-0.42735
	(0.3389)
**Enterobacterales; Enterobacteriaceae; *Plesiomonas***	**0.77155**
	**(0.0422)**
**Lactobacillales; Streptococcaceae; *Lactococcus***	-0.45660
	(0.3031)
**Enterobacterales; Enterobacteriaceae; *Edwardsiella***	0.24193
	(0.6012)
**Bacteroidales; Bacteroidaceae; *Bacteroides***	-0.53274
	(0.2183)
**Enterobacterales; Morganellaceae; *Morganella***	-0.63103
	(0.1286)
**Clostridiales; Peptostreptococcaceae; *Paraclostridium***	0.55760
	(0.1934)

*p-value < 0.05

## 4. Discussion

From 1973, The Nurmi concept or competitive exclusion has been developed [34]. This concept considers that the administration of intestinal microbiota from an adult animal to young animals could give protection against pathogenic bacteria [35]. In the present study, we developed a CFCEC with antibacterial activity against *S*. *agalactiae*. It was maintained in TSB medium reported in the successful development of a competitive exclusion culture from different animals including fish culture [36,37]. This medium is relatively inexpensive, easily obtainable and easily reproducible from off-the-shelf-ingredients, also appropriate to support the growth of aerobic and anaerobic bacteria. We did not intend to use complex customized media to mimic intestinal conditions of the fish intestine as it will not have the aforementioned advantages, important in foreseen scaling-up applications, and TSB has proven to be suitable medium for the purposes of quickly enriching consortia from intestinal homogenates of *O*. *niloticus* allowing the isolation of bacteria with probiotic activity [37].

Nisbet et al. [38] used a continuous flow apparatus to develop a competitive exclusion culture against the enteropathogenic bacteria *Salmonella* Typhimurium. A commercial competitive exclusion culture, Preempt, was developed under that methodology [39]. The genotypic characterization of Preempt by Wagner [40] showed that this CEC is composed of 29 bacteria of the genera *Stenotrophomonas*, *Bacteroides*, *Acinetobacter*, *Enterococcus*, *Lactococcus*, *Lactobacillus*, *Eubacterium*,and *Pediococcus*. According to Nisbet et al. [41], after 6 days of fermentation, where the production of bacteriocins, volatile fatty acids and the competition for nutrients ensure the survival of bacteria with antibacterial activity against pathogens, a CFCEC reaches steady state, exhibiting stable pH, optical density and viable cell count. In the present study, the CFCEC for tilapia reached steady state after the 9 days according to the Baranyi and Roberts predictive model of aerobic and anaerobic bacteria plate count on TSA. However, a denaturing gradient gel electrophoresis (DGGE) analysis over different days of the CFCEC let us know that there were some changes in bacterial composition over time (Fig 7).

Given the hints obtained from the DGGE analysis, Illumina sequencing was performed in order to precisely identify the bacterial types present in the tilapia specimens and derived at different times of the CFCEC fermentation. According toAdeoye et al. [42], the orden *Clostridiales and Aeromonadales*, *the clase Gammaproteobacteria*, *and the genera Cetobacterium sp*., *Aquaspirillum* sp., *Edwardsiella* sp. and *Plesiomonas* sp. are the main bacteria in the intestinal tract of tilapia. in the intestinal tract of tilapia. In our study, samples from adult fishes from two farms in different geographical regions of Colombia were used to establish the CFCEC and the samples were subjected to characterization by bacterial 16S rRNA gene amplicon Illumina sequencing. From that result it was observed that fish from the two farms showed variation in the intestinal bacteria composition. According to Li et al. [43], this variation in the composition could be affected by the feeding habits such as water quality, growing densities, physicochemical parameters, among others factors. For that reason, herbivorous fishes are dominated by cellulose-degrading bacteria such as *Clostridium*, *Citrobacter* and *Leptotrichia*, while in carnivorous fish the most abundant bacteria are *Cetobacterium* or *Halomonas* [44]. In our results, interestingly, fishes from Atlantic farm were dominated by *Clostridium*, while the samples from Llanos farm fishes were mainly composed of *Cetobacterium* (Fig 3).

Anaerobic bacteria such as *Cetobacterium* and *Clostridium* are described producers of volatile fatty acids, vitamins and enzymes (lipase, amylase, protease and casease) important in fish nutrition [45]. The most abundant bacterium in the first days of the CFCEC, was *Cetobacterium* sp., a Gram-negative micro aerotolerant rod [46] and non-spore-forming [47]. This bacterium has been found in most freshwater fishes [48]. *Cetobacterium*, is a vitamin B12-producer and excretes butyrate as fermentation product from carbohydrates and peptides [49,50]. Butyric acid has been reported as an inhibitor of freshwater pathogens (*Vibrio* sp., *Aeromonas* sp., *Flavobacterium* sp., and *Yersinia* sp.) [46]. Acetic and propionic acids are other products of the peptone and carbohydrates fermentation [51]. The second major observed genus was *Clostridium* which includes species with saccharolytic, fiber-fermenting and proteolytic activities [49]. *Clostridium* spp. are mainly present in plant-fed fish because this genus is known as cellulose-degrading bacteria [43].

The analyses of the 16S rRNA gene amplicons sequencing revealed bacterial compositional changes in the CFCEC upon incubation time. One of the major changes observed was the decrease in relative abundances of the *Cetobacterium* from the second day. According to Hao et al. [51] an increase in the population of *Bacteroides*, and the families Lachnospiraceae and Erysipelothrichaceae might be linked to decreases in the populations of *Cetobacterium somerae*. Our results showed that higher populations of *Bacteroides* and *Lachnospiraceae* are also related to *Cetobacterium* decreases at day 33 of the reactor fermentation. *Bacteroides* have a reported role in the fermentation of plant-derivate substrates producing short-chain fatty acids such as acetate or succinate [49], and *Lachnospiraceae* produce acetate, butyrate and propionate from complex polysaccharides [52], which differ with *Cetobaterium* that ferment animal-derivate substrates as previously described. Finally, *Bacteroides* and *Lachnospiraceae* are interesting bacteria that must be studied for their roles as probiotic bacteria in fish intestines including the modulation of the intestinal microbiota.

Another change in the CFCEC was the increase in the relative abundance of lactic acid bacterial types. *Lactococcus* was the most abundant genus at 33 days of the CFCEC. However, despite the well documented antibacterial effects of *Lactococcus*, medium from the 33-day CFCEC did not exhibit agar diffusion anti-*S*. *agalactiae* activity. *Lactococcus* is a Gram-positive spherical-shaped bacterium [53]. That is generally recognized as safe (GRAS), and therefore commonly used in food industry [54]. In fish, there are some evidences of the probiotic potential of this genus such as growth promotion, antibacterial activity and immunomodulation [55]. *L*. *lactis* has improved growth rate in several culture fish such as *Epinephelus coioides*, *Epinephelus bruneus*, *Rachycentron canadum*, *O*. *niloticus*, *Labeo rohita* and *Pagrus major* [56]. *L*. *lactis* is a nisin-producing bacterium showing antibiotic activity against *Streptococcus iniae*, *Streptococcus parauberis*, *Enterococcus viikkiensis*, and *Lactococcus garviae* [53,57]. Finally, *Lactococcus* have been applied to salmon and turbot as a probiotic because of its immunomodulatory properties [58,59].

Even though most of the bacteria cultured in the CFCEC were commensal bacteria, there were some fish pathogens that were present in the intestinal contents used to develop the CFCEC, among them *Edwardsiella* (3% of the total reads), *Aeromonas* (0,2% of the total reads), *Plesiomonas* (10% of the total reads) and *Streptococcus* (0.001% of the total reads) (Fig 5). *Edwardsiella* are Gram-negative bacteria causing edwardsiellosis under imbalanced environmental conditions such as high water temperature and hypoxia [60]. *A*. *hydrophila* is a Gram-negative coccobacillus, an anaerobic facultative inhabitant of aquatic environments and also found in the gastrointestinal tract of aquatic and terrestrial animals, and could also cause diseases in lower vertebrates such as freshwater fishes [61]. *Plesiomonas* is a common member of the microbiota of freshwater fishes. There are reports that show *Plesiomonas* as a pathogenic bacteria, however, the most important concern in this genus is a public health risk to consumers because it has been associated with enteric diseases in humans [62,63]. Other infectious agents associated with fish mortalities are the Gram-positive pathogenic strains of the genus *Streptococcus* [64]. The presence of those kind of opportunistic bacteria is normal in fish gastrointestinal tract, however, change in environmental conditions such an increase in water temperature and decrease in dissolved oxygen produce stress on fish allowing the pathogenic bacteria to cause the diseases symptoms; for that reason it is necessary to offer strategies for maintaining the stability of the gut microbiota [65]. In this study, it was possible to observe that bacteria present in the CFCEC have the capability of decreasing the abundances of the opportunistic pathogenic bacteria during the incubation time, as the Illumina sequencing analysis showed, decreasing from 10% to 2.8% *Plesiomonas*, from 3% to 0.8% *Edwardsiella*, from 0.2 to 0.01% *Aeromonas* and *Streptococcus* kept low after 33 days of the CFCEC (Fig 5).

There are different antimicrobial mechanisms ascribed to competitive exclusion which are biological (low-level of oxygen), chemical (organic acids), biochemical (antimicrobial substances), nutritional (competition for nutrients) [66]. In the first three days, the cell-free extracellular products of the CFCEC exhibited an antibacterial activity against *S*. *agalactiae*. Given this result, we consider it is very likely that the CFCEC harbored bacterial types able to produce antibacterial substances against this fish pathogen. Given the loss of antibacterial activity from day three in the culture supernatant, as determined by agar diffusion results, it is important to evaluate a competitive exclusion and displacement of pathogens by the CFCEC consortium in a chemostat or by *in vivo* challenging of fishes in order to identify whether other mechanisms are involved in the inhibition of pathogenic bacteria by the CFCEC derived from tilapia gut microbiomes.

As main conclusions of this study are: 1) Nile tilapia intestinal microbiota in CFCEC demonstrated *in vitro* antimicrobial potential against the fish pathogen *S*. *agalactiae*; and 2) 16S rRNA gene amplicon sequencing revealed the abundant bacterial types at different stages of the CFCEC establishment, with compositional changes observed over time that were related to the increase or decrease of anti-*Streptococcus agalactiae* activity. Additional characterization of the CFCEC is needed to evaluate the competitive exclusion and displacement potential against *S*. *agalactiae* and to identify the mechanisms used by the probiotic bacteria in order to inhibit the growth of pathogenic bacteria. For further *in* vivo studies, it will be necessary to develop a CFCEC culture selecting bacteria based on probiotic properties and metabolic compatibility.

## Supporting information

S1 Fig Agar diffusion test of the extracelullar products at 0, 1, 2, 3, 4, 5, 6, 7, 8, 9, 11, 12, 13, 14, 15, 17, 18, 24, 28, 33, 35, 42, 45, 54 and 56 days of the continuous-flow competitive exclusion culture. (TIF)

S2 Fig Diameters of the zones of inhibition of the extracellular products at day 0, 1 and 2 of the continuous-flow competitive exclusion culture. (TIF)
