# Supplementary figures and images for: Establishment and characterization of a competitive exclusion bacterial culture derived from Nile tilapia (Oreochromis niloticus) gut microbiomes showing antibacterial activity against pathogenic Streptococcus agalactiae
Source: PLoS One. 2019 May 3;14(5):e0215375. doi: 10.1371/journal.pone.0215375 (PMC6499431; doi:10.1371/journal.pone.0215375)

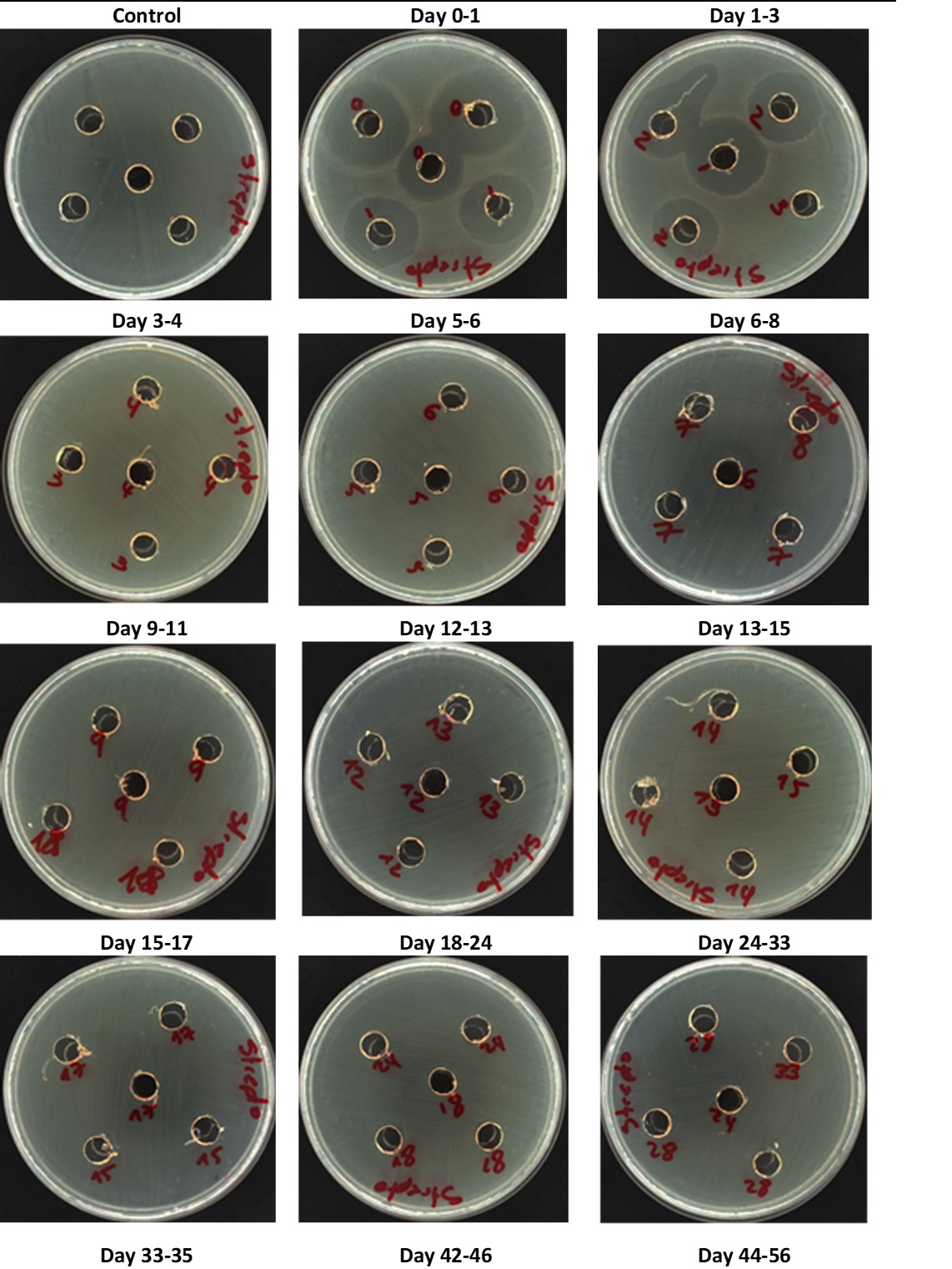

Supplement: S1 Fig — (TIF) [file pone.0215375.s001.tif]

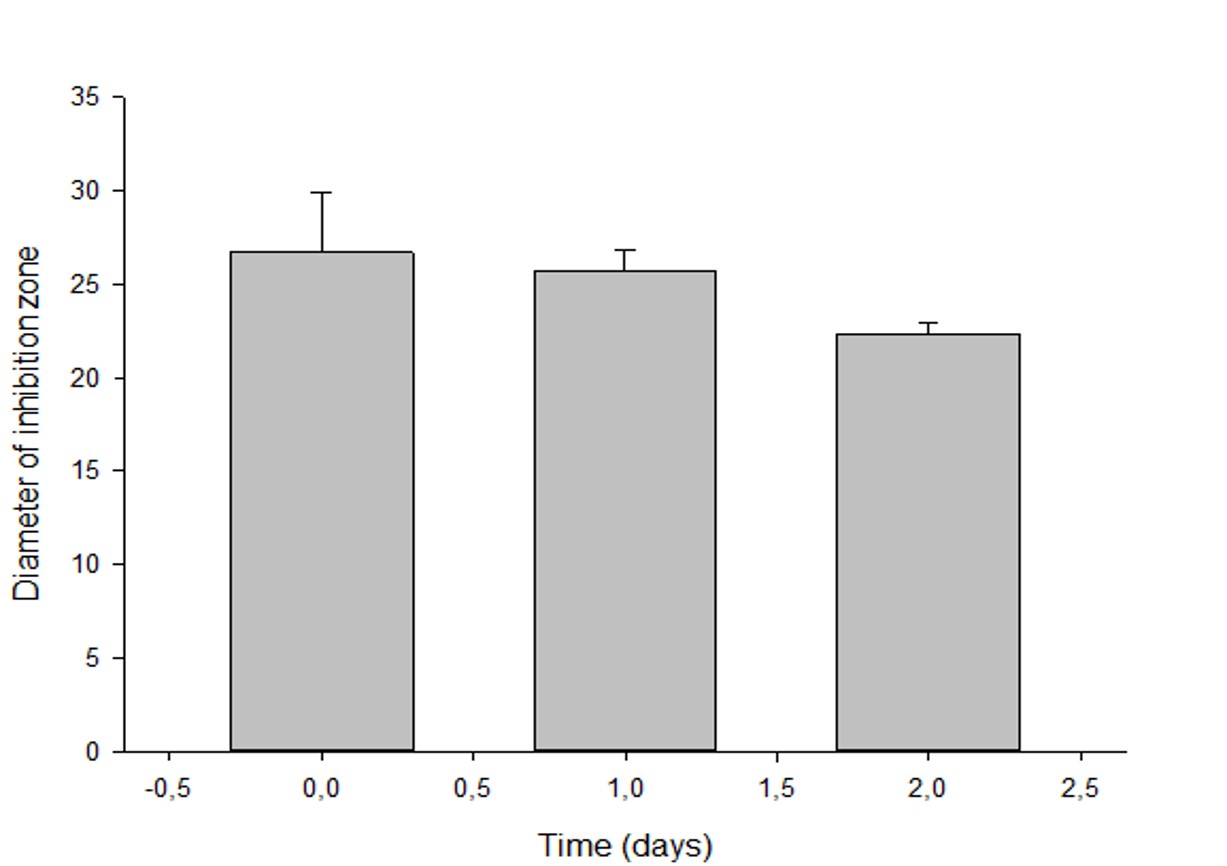

Supplement: S2 Fig — (TIF) [file pone.0215375.s002.tif]
